# 2148. *In vitro* Potency of a Leucyl-tRNA Synthetase Inhibitor, MRX-6038, Against *Mycobacterium tuberculosis*, *Mycobacterium avium*, and *Mycobacterium abscessus*

**DOI:** 10.1093/ofid/ofad500.1771

**Published:** 2023-11-27

**Authors:** Carolyn M Shoen, Michelle S DeStefano, Wen Wang, Michael H Cynamon

**Affiliations:** Veteran's Health Research Institute, Syracuse, New York; Veteran's Health Research Institute, Syracuse, New York; MicuRx Pharmaceuticals, Inc., Foster City, California; Veteran’s Health Research Institute, Syracuse, New York

## Abstract

**Background:**

The family of Mycobacteriaceae contains hundreds of species, some causing diseases in humans. The most well-known is *Mycobacterium tuberculosis* (Mtb), the causative agent for tuberculosis. Approximately 2 billion people are infected with *M. tuberculosis*. Drug-resistant strains of *M. tuberculosis* have emerged in the last 30 years, making the development of newer, effective drugs important. Non-tuberculous mycobacteria (NTM) cause difficult to treat infections. *M. abscessus* (Mabs) and *M. avium* (MAC) fall within this category. Drug-resistance and/or drug tolerance is a serious hurdle in trying to control NTM infections. The goal of this study was to evaluate the *in vitro* activity of a novel leucyl-tRNA synthetase inhibitor, MRX-6038, against Mtb, MAC, and Mabs.

**Methods:**

MRX-6038, epetroborole (EBO), and GSK656 (GSK) was provided by MicuRx Pharmaceutical Co., Ltd. Clarithromycin (CLR) and isoniazid (INH) were purchased from Biosyth Ltd and Sigma-Aldrich Chemical Company, respectively. Stock solutions were dissolved in 100% DMSO and frozen at -20^o^C until used. Drugs were serially diluted in cation adjusted Mueller-Hinton broth (CAMHB) (Mabs), CAMHB with 5% OADC (MAC), and 7H9 Broth with 10% OADC (Mtb) in microtiter plates for MIC evaluation in duplicate. Wells were inoculated with ∼ 1.25 x 10^5^ CFU/ml of bacteria. Plates were incubated for 4-5 (Mabs), 7 (MAC), or 14 (Mtb) days at 37^o^C in ambient air. The MIC is defined as the lowest concentration of drug that yielded no visible turbidity.

**Results:**

The MIC_50_ and MIC_90_ (µg/ml) of the MAC isolates against CLR, EBO, and MRX-6038 were 1 and 4, 1 and 8, and 1 and 8, respectively. The MIC_50_ and MIC_90_ of the Mabs isolates against CLR, EBO, and MRX-6038 were 8 and 16, 0.125 and 0.25, and 0.25 and 0.25, respectively. The MIC_50_ and MIC_90_ of the Mtb isolates against INH, GSK, and MRX-6038 were 1 and 8, 0.06 and 0.06, and 0.5 and 1, respectively. Many of the Mtb isolates used were known to be MDR strains.

**Figure:**
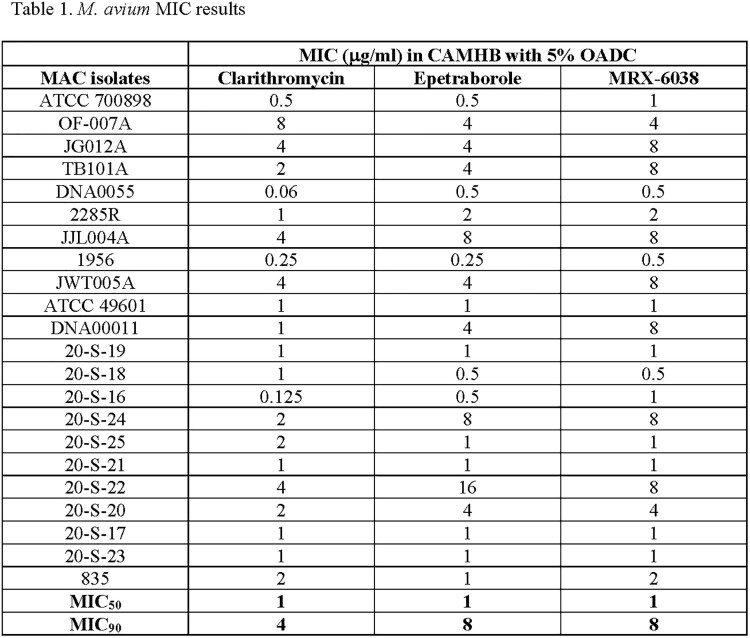

**Figure:**
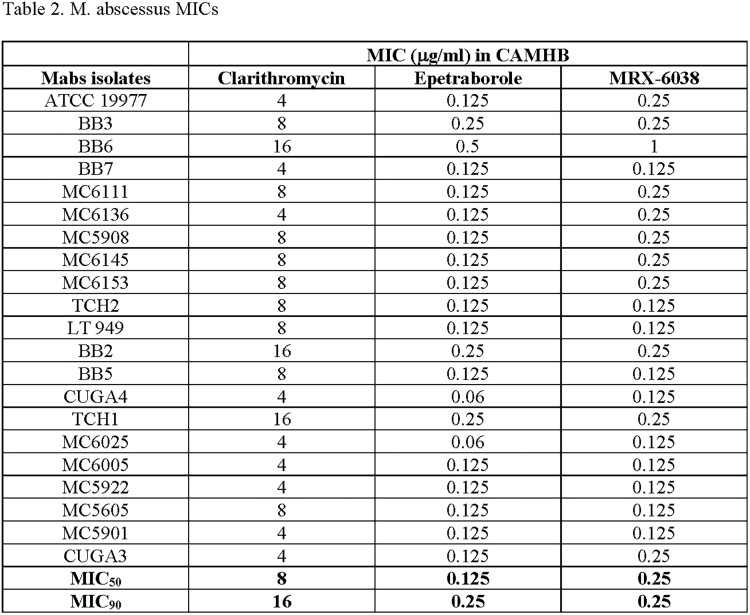

**Figure:**
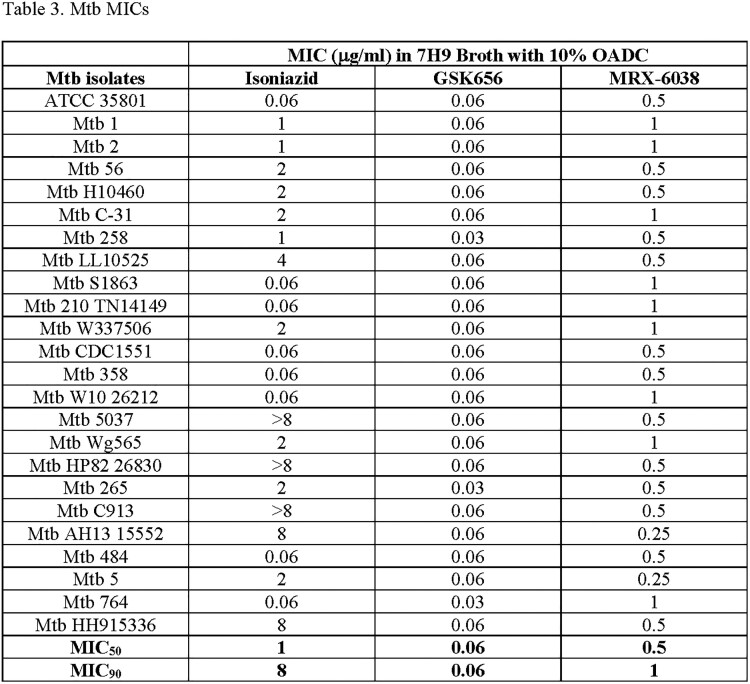

**Conclusion:**

The *in vitro* activity of MRX-6038 was quite promising against Mabs, MAC, and Mtb. Based on work by others, MRX-6038 has shown encouraging *in vivo* activity against Mabs in mice. The next steps will be to determine the *in vivo* activity against both MAC and Mtb infection in mice.

**Disclosures:**

**Wen Wang, PhD**, MicuRx Pharmaceuticals: Employee of

